# Population Structure Assessed Using Microsatellite and SNP Data: An Empirical Comparison in West African Cattle

**DOI:** 10.3390/ani11010151

**Published:** 2021-01-11

**Authors:** Isabel Álvarez, Iván Fernández, Amadou Traoré, Nuria A. Menéndez-Arias, Félix Goyache

**Affiliations:** 1 Servicio Regional de Investigación y Desarrollo Agroalimentario, E-33394 Gijón, Spain; ialvarez@serida.org (I.Á.); ifernandez@serida.org (I.F.); nuriamendezarias@hotmail.com (N.A.M.-A.); 2 Institut de l’Environnement et des Recherches Agricoles (INERA), Ouagadougou 04 BP 8645, Burkina Faso; traore_pa@yahoo.fr

**Keywords:** West African cattle, clustering, population structure, geographical projection, synthetic maps, genetic variability

## Abstract

**Simple Summary:**

Projection of genetic variability on geographic maps is a useful strategy to ascertain population structure and gene flow events when previous genetic information on the scenarios analyzed is not high. Here, we compared the performance of microsatellite sets and Single Nucleotide Polymorphism (SNP) arrays to identify the population structure and between-populations identity in a sample of West African cattle. Large SNP arrays were superior in detecting the population structure due to a more precise assessment of genotypic information of the individuals. However, the projection of genetic parameters on geographical maps was comparable between the SNP and microsatellite data. Geographic-based analyses of genetic variation areuseful inavoiding overinterpretation of the results obtained. Microsatellite markers can still be useful, particularly if the research focuses on non-model organisms or if either the funding or the availability of efficient hardware and software to handle large datasets is limited.

**Abstract:**

A sample of 185 West African cattle belonging to nine different taurine, sanga, and zebu populations was typed using a set of 33 microsatellites and the BovineHD BeadChip of Illumina. The information provided by each type of marker was summarized via clustering methods and principal component analyses (PCA). The aim was to assess differences in performance between both marker types for the identification of population structure and the projection of genetic variability on geographical maps. In general, both microsatellites and Single Nucleotide Polymorphism (SNP) allowed us to differentiate taurine cattle from zebu and sanga cattle, which, in turn, would form a single population. Pearson and Spearman correlation coefficients computed among the admixture coefficients (fitting K = 2) and the eigenvectors corresponding to the first two factors identified using PCA on both microsatellite and SNP data were statistically significant (most of them having *p* < 0.0001) and high. However, SNP data allowed for a better fine-scale identification of population structure within taurine cattle: Lagunaire cattle from Benin were separated from two different N’Dama cattle samples. Furthermore, when clustering analyses assumed the existence of two parental populations only (K = 2), the SNPs could differentiate a different genetic background in Lagunaire and N’Dama cattle. Although the two N’Dama cattle populations had very different breeding histories, the microsatellite set could not separate the two N’Dama cattle populations. Classic bidimensional dispersion plots constructed using factors identified via PCA gave different shapes for microsatellites and SNPs: plots constructed using microsatellite polymorphism would suggest the existence of weakly differentiated, highly intermingled, subpopulations. However, the projection of the factors identified on synthetic maps gave comparable images. This would suggest that results on population structuring must be interpreted with caution. The geographic projection of genetic variation on synthetic maps avoids interpretations that go beyond the results obtained, particularly when previous information on the analyzed populations is scant. Factors influencing the performance of the projection of genetic parameters on geographic maps, together with restrictions that may affect the election of a given type of markers, are discussed.

## 1. Introduction

The availability of SNP arrays including thousands of markers has the potential to address questions in population genetics such as the evaluation of distance among population, diversity, and structuring, with a higher resolution than that previously obtained with microsatellites due to increased precision and smaller confidence intervals around diversity measures [1].

The performance of microsatellite sets and SNP arrays has mainly been compared in non-model organisms. Although the magnitude of the differentiation metrics can be quite different, estimates of the between-populations’ genetic distances obtained using either microsatellites or SNPs generally have a strong correlation [1,2,3]. This may not be the same for within populations diversity estimates computed using microsatellites that may not adequately reflect the genome-wide genetic diversity estimated from SNPs, particularly if the size of the microsatellite set used is small [3]. Therefore, inferences on population prioritization for conservation, genotype-fitness correlation, and others needing individual-level genotype information may vary depending on the marker used [2,4]. Furthermore, although most examples suggest that SNP arrays are more informative to identify further sub-structuring [3], it is admitted that patterns of population structure based on either microsatellites or SNPs are usually in accordance [1]. In any case, the contrary has also been reported [5].

Although clustering methods are informative on the existence of hidden population structuring and differentiation within a sample, geographical projection of parameters summarizing genetic variation on synthetic maps is useful to shed light on the causes and patterns of the differences assessed [6,7]. Projection of genetic diversity on geographical maps allows for the identification ofdifferent geographical scenarios such as local spots of (either higher or lower) genetic diversity or scenarios of spatial smoothing of allele frequencies between populations [8,9]. When no geographical constraints to gene flow exist, it is assumed that populations are related to each other via isolation-by-distance processes and, therefore, sampling of all populations is not required [10]. However, a possible effect of the nature of markers used on the assessment of geographical patterns of genetic variation has not been tested thusfar.

Here, we typed 185 cattle sampled in three different West African countries with a set of microsatellite markers and SNP arrays to compare the performance of clustering methods across datasets and to ascertain whether a geographical assessment of genetic variation derived from either microsatellite or SNP polymorphisms is valid as a reflection of genetic variation and differentiation.

## 2. Materials and Methods

### 2.1. Samples and Genotyping

A total of 185 blood samples (44 corresponding to males) were obtained from taurine (*Bos taurus*; 105), zebu (*B. indicus*; 44), and sanga (36) cattle individuals belonging to eight cattle populations of Benin, Burkina Faso, and Congo (Table 1). The N’Dama cattle sampled in Congo derived from two N’Dama bulls and 40 N’Dama heifers imported from the Fouta-Djallon region of Guinea [11], and therefore were assigned to their original geographical coordinates. Morphology and breeding scenarios of the populations sampled were previously described [11,12,13]. Total DNA was isolated from blood samples following standard procedures [14].

Thirty-three microsatellites (AGLA293, BM2113, BM2504, BM6526, BM757, BMS2626, BMS356, BMS975, CP34, CSRM60, CSSM015, CSSM43, CSSM66, ETH10, ETH225, ETH3, ILSTS005, ILSTS006, ILSTS008, ILSTS011, ILSTS023, ILSTS028, ILSTS033, ILSTS036, ILSTS050, McM53, MGTG4B, RBP3, SPS113, TGLA048, TGLA122, TGLA126, TGLA227) were analyzed for all samples. Twenty-one of these microsatellites were previously used to characterize contributions to diversity in cattle [15]. Genotyping was performed on an Automatic Sequencer ABI 310 (Applied Biosystems, Barcelona, Spain).

The whole dataset was also typed using the BovineHD BeadChip of Illumina (Illumina Inc., San Diego, CA, USA; 777,962 SNPs) following standard protocols. SNP coordinates were mapped on the bovine UMD 3.1 reference genome assembly. The software GenomeStudio (Illumina Inc., San Diego, CA, USA) was used to generate standard .ped and .map files. Sample and marker-based quality control measures were performed using the program PLINK V 1.9 [16]. A GenCall score cutoff of 0.15 and average sample call rate of 99% were considered. All unmapped SNPs, those mapping to sexual chromosomes, SNPs with a genotyping rate lower than 90%, and those below a minor allele frequency threshold of 0.05 were removed. To avoid departures from Hardy–Weinberg proportions due to genotyping errors, SNPs that did not pass the Hardy–Weinberg test for *p*  ≤  0.001 were also removed. A total of 543,595 SNPs located on the 29 bovine autosomes passed the quality control for the whole sample analyzed. 

### 2.2. Population Structure Analyses

Principal component analysis (PCA) was performed on microsatellite allelic frequencies, according to the recommendations by Cavalli-Sforza et al. [17] using the Proc Factor of the statistical package SAS/STAT (SAS Institute, Cary, NC, USA). Furthermore, the program STRUCTURE [18] was run on the individual genotypes, under the admixture model and considering correlated allele frequencies, to ascertain cryptic genetic structure in the microsatellite dataset. The most likely number of clusters (K) in the dataset was identified using the STRUCTURE HARVESTER v.0.6.8 website [19]. K was set to vary between oneand eight, and 10 simulations with different starting points for each K-value. All runs used burn-in periods of 100,000 iterations and data collection periods of 1,000,000 iterations.

The program PLINK V 1.9 [16] was used to compute PCA on the SNP array genotypes. Furthermore, clustering analysis was carried out using the program ADMIXTURE v1.23 [20,21]. This program calculates the maximum likelihood estimates of individual ancestries based on data provided by multiple loci using a similar algorithm than STRUCTURE, but being computationally much faster. Analyses were conducted for 1 ≤ K ≤ 8 withK the number of clusters given in the data. The optimal number of clusters was determined via cross-validation as the value of K exhibiting a lower cross-validation error compared to other K values. The dataset was divided into five folders for each K. Folders were sequentially used as test sets while the other four were used for training.

### 2.3. Information Summary and Projection on Synthetic Maps

Using either microsatellites or SNP genotyping data, eigenvectors computed for each individual via PCA were used to construct dispersion plots, and the 75% confidence interval of the dispersion of the individuals per population using the library ggplot2 of R (http://CRAN.R-project.org/) [22].

Boxplots summarizing the information provided by the individual admixture coefficient $\hat{q}$ estimated for each individual with STRUCTURE and ADMIXTURE and K = 2 were also constructed using the library ggplot2 of R (http://CRAN.R-project.org/). Using this approach, $\hat{q}$ estimates inform on the amount of an individual’s genome that would be derived, assuming two parental populations only. Individuals with $\hat{q}$ values ranging from 0 to 0.1 or from 0.9 to 1.0 were assumed to belong to a parental population [23,24]. As aconsequence, hybrid individuals would be those with $\hat{q}$ estimates ranging from 0.1 to 0.9. Note that if one population is not derived from those expected to be parental, the individuals belonging to the analyzed populations can obtain intermediate or extreme $\hat{q}$ values at random.

Pearson and (rank) Spearman correlation coefficients between the individual coefficients $\hat{q}$ estimated for K = 2 and eigenvectors computed for each individual via PCA were computed using the Proc Corr of SAS/STAT.

Admixture coefficients estimated for K = 2 and PCA eigenvectors computed for each individual were also used to construct interpolation maps drawn using the Spatial Analyst Extension of the program ArcView. The inverse distance weighted (IDW) option with a power of two was selected for the interpolation of the surface. IDW assumes that each input point has a local influence that diminishes with distance. The area of sampling of each breed was used as the geographic coordinates, and the six nearest neighbors were used for the calculation. Interpolation surfaces were divided into eight equal classes.

## 3. Results

**Figure 1:**
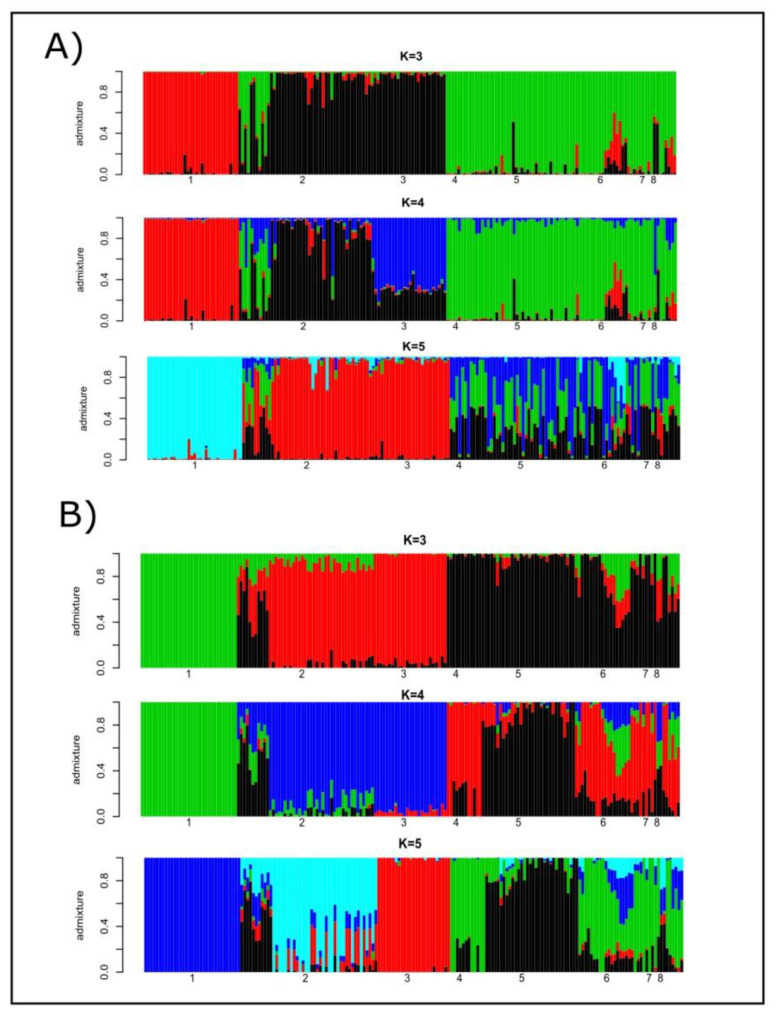
Barplots of individual ancestries for K = 3, K = 4 and K = 5 estimated using the program STRUCTURE on microsatellite genotypes (Plot **A**) and the program ADMIXTURE v1.23 on SNP polymorphism (Plot **B**). Numbers mean the following: (1) Lagunaire; (2) N’Dama (Burkina Faso); (3) N’Dama (Congo); (4) Zebu Peul (Benin);(5) Zebu Peul (Burkina Faso); (6) Borgou; (7) Lobi; and (8) Zou. Populations from 1 to 3 belong to West African taurine cattle, populations 4 and 6 belong to West African zebu cattle, and populations from 6 to 8 belong to sanga cattle.

Cryptic genetic structure was assessed using the programs STRUCTURE and ADMIXTURE. The most likely number of K estimated using microsatellites was four, while that estimated using SNP genotypes was three (Supplementary Figure S1). When microsatellites were considered, the number of clusters was equally likely for K = 5. In the case of SNPs, K = 3 and K = 5 had comparable cross-validation errors. Figure 1 illustrates the individual ancestries estimated from K = 3 to K = 5 using microsatellite polymorphism (Plot A) and SNP data (Plot B). Microsatellite- and SNP-based results followed similar patterns: (a) Lagunaire cattle formed their own cluster; (b) the two N’Dama cattle populations shared ancestry; and (c) zebu cattle were the main source of genes for the sanga cattle. The main difference between markers was that the more likely number of K for microsatellites (K = 4) accounted for the different breeding histories of the two N’Dama populations sampled. Regarding SNPs, this only occurred for K = 5. However, the cross-validation errors computed for K = 3 (the most likely) and K = 5 were the same for any practical purpose (Figure S1). The SNP data tended to differentiate ancestries for sanga (Borgou, Lobi, and Zou) cattle to a higher extent than microsatellite polymorphism.

**Figure 2:**
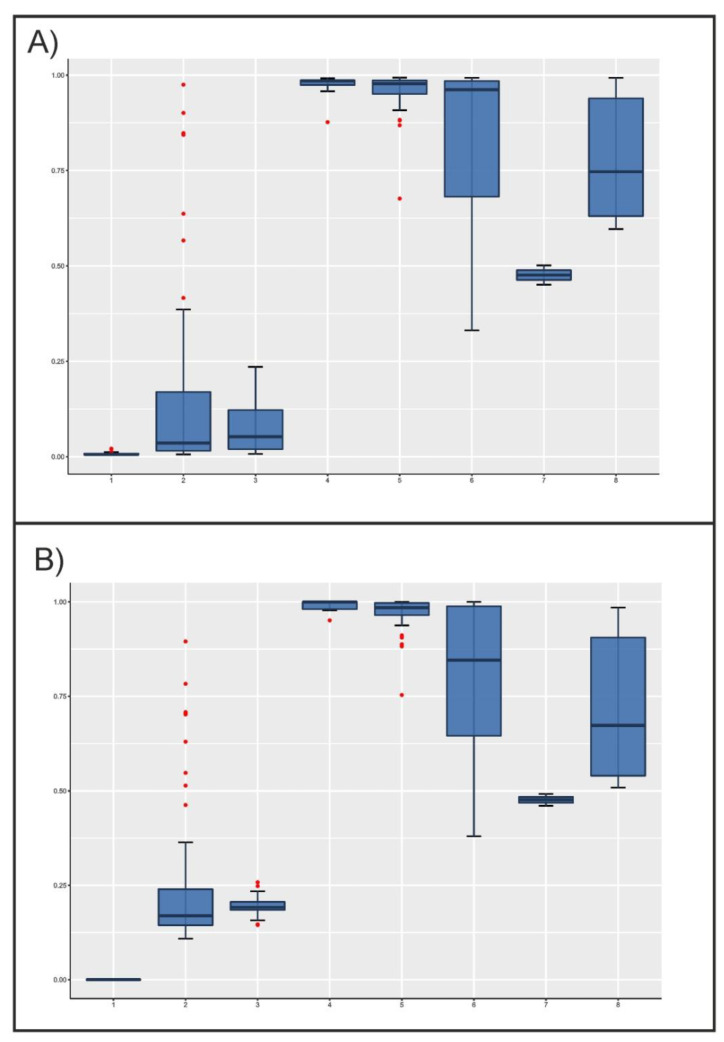
Boxplots illustrating the variation of individual admixture coefficient, $\hat{q}$q, estimated using microsatellites (Plot **A**) and SNPs (Plot **B**), by cattle population assuming K = 2. The box represents the range that contains the values within the limits of the standard error of the mean, the line within the box indicating the mean value. The whiskers are the lines that extend from the box to the standard deviation excluding outliers. Outliers, which are represented by red dots, are values that are 1.5–3 standard error lengths from the upper or lower edge of the box. Numbers on the *X*-axis mean the following: (1) Lagunaire; (2) N’Dama (Burkina Faso); (3) N’Dama (Congo); (4) Zebu Peul (Benin); (5) Zebu Peul (Burkina Faso); (6) Borgou; (7) Lobi; and (8) Zou. Populations from 1 to 3 belong to West African taurine cattle, populations 4 and 6 belong to West African zebu cattle, and populations from 6 to 8 belong to sanga cattle.

When software used for structure analyses was forced to assume the existence of two parental populations only (K = 2), the posterior distributions of admixture proportions of the individuals analyzed were not uniform (Figure 2). Although the Lagunaire taurine cattle had asmaller dispersion of $\hat{q}$ values, the zebu cattle populations showed narrower distributions than taurine cattle, the former with $\hat{q}$ values higher than 0.9. Although most taurine cattle would belong to the same parental population regarding microsatellites ($\hat{q}$ values lower than 0.1; Figure 2A), SNPs would suggest that N’Dama cattle would belong to a different parental population than Lagunaire cattle. In any case, the N’Dama cattle of Burkina Faso showed a wide distribution and a considerable number of extreme values near the sanga and zebu cattle individuals. As expected, sanga cattle mainly took intermediate and widely distributed $\hat{q}$ values regardless of the markers considered. However, their $\hat{q}$ values were nearer to those of zebu cattle, except for the two Lobi individuals (Figure 2).

**Figure 3:**
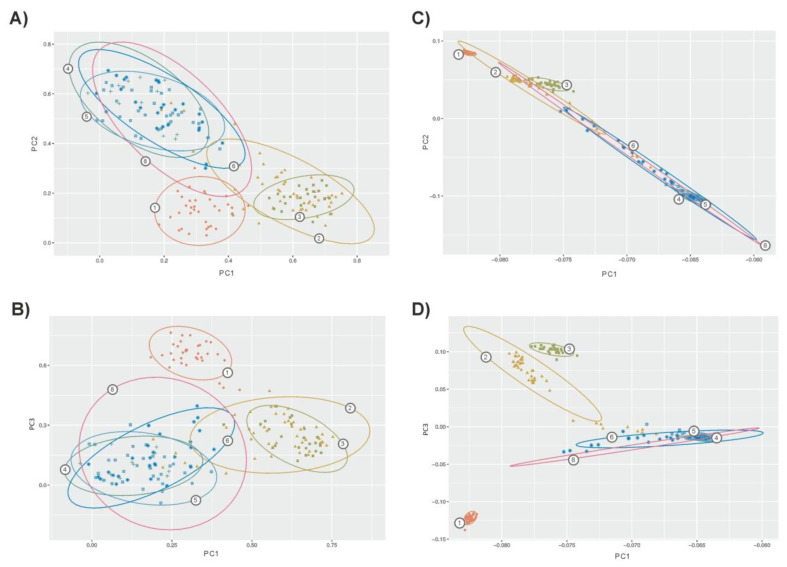
Bidimensional plots illustrating dispersion of the cattle individuals sampled according to the three first factors computed via PCA on microsatellite data (Plots **A**,**B**) and SNPs (Plots **C**,**D**). Plots A and C wereconstructed using Factors 1 (on *X*-axis) and 2; Plots B and D wereconstructed using Factors 1 (on *X*-axis) and 3. Contour plots illustrate the 75% confidence region of the relationships betweenthe individuals assigned to each population. Numbers on contours mean the following: (1) Lagunaire; (2) N’Dama (Burkina Faso); (3) N’Dama (Congo); (4) Zebu Peul (Benin); (5) Zebu Peul (Burkina Faso); (6) Borgou; and(8) Zou. Populations from 1 to 3 belong to West African taurine cattle, populations 4 and 6 belong to West African zebu cattle and populations 6 and 8 belong to sanga cattle. Confidence intervals for the two Lobi individuals were not computed.

Using microsatellites, PCA allowed us to identify 39 factors with an eigenvalue higher than 1 explaining 79% of the genetic variability. In total, the three first factors explained 44% of variability (32%, 8.3%, and 3.7%, respectively). Using SNPs, PCA allowed us to identify eight factors with an eigenvalue higher than 1, explaining 21% of the genetic variability. The three first factors identified explained 17% of variability (11%, 4%, and 2%, respectively). In general, PCA confirmed the general scenario depicted by genetic structure analyses. However, microsatellites (Figure 3A,B) gave a lower differentiation among populations with clear overlap between confidence intervals for the dispersion of the individuals assigned to each population. This was particularly true for the zebu and sanga cattle populations. Within taurine cattle, Lagunaire individuals tended to be separated. SNP-based PCA (Figure 3C,D) gave clearer separation of the taurine cattle from the zebu and sanga cattle. Furthermore, the confidence intervals computed separated the two N’Dama populations sampled. In any case, the figure constructed using the two more informative factors computed on SNPs (Plot 3C) suggested the existence of “continuous” genomic variation between taurine and zebu West African cattle.

Pearson and Spearman correlation coefficients computed among the $\hat{q}$ values estimated for each individual assuming two parental populations only (K = 2) and the eigenvectors corresponding to the first three factors identified using PCA on both microsatellite and SNP data are given in Table 2. The admixture coefficients estimated using microsatellites and SNPs had high product-moment (ρ = 0.978) and rank (ρs = 0.947) correlation coefficients (*p* < 0.0001). This could also be assessed for the second factor identified using either microsatellites or SNPs (ρ = −0.905 and ρs = −0.836). Furthermore, both Factor 2s, separating the taurine populations from the other cattle populations assessed (Figure 3), had strong and negative Pearson and Spearman correlation coefficients with admixture coefficients (ranging from ρ = 0.912 to ρs = −0.995), suggesting that they may give the same information in practical terms. Non-significant Pearson and Spearman correlation coefficients are related to the less informative factors (Factor 3s) identified on both datasets.

**Figure 4:**
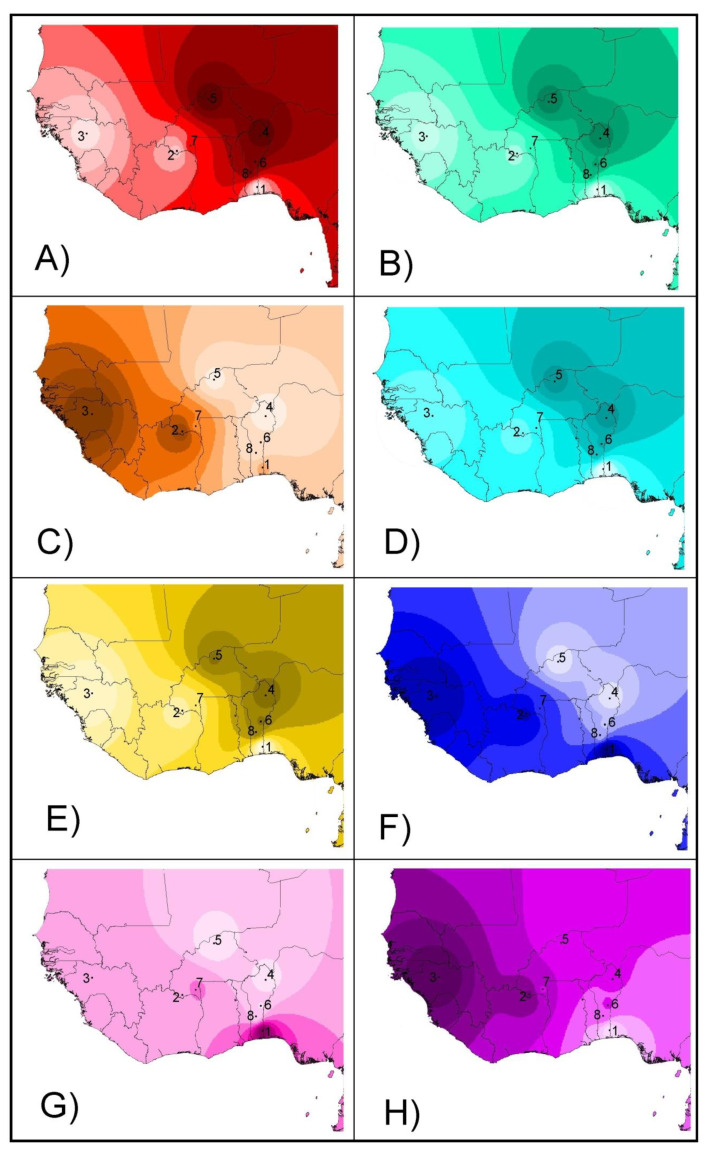
Synthetic maps illustrating the geographical variation of the individual admixture ($\hat{q}$) estimated on microsatellite (Plot **A**) and SNP (Plot **B**) variation assuming K = 2. The same maps were constructed using the first (Plots **C**,**D**), second (Plots **E**,**F**), and third (Plots **G**,**H**) factors identified on the microsatellite (left column) and SNP (right column) variation. Colors should be interpreted as differences between mean $\hat{q}$ coefficients or eigenvalues: higher valuesare darker. Points illustrate the geographical coordinates in which the breeds were considered to be sampled. The IDW option of the Spatial Analyst Extension of the program ArcView was fitted to a power of two assuming that each input point has a local influence that diminishes with distance. Numbers mean the following: (1) Lagunaire; (2) N’Dama (Burkina Faso); (3) N’Dama (Congo); (4) Zebu Peul (Benin); (5) Zebu Peul (Burkina Faso); (6) Borgou; (7) Lobi; and (8) Zou. Populations from 1 to 3 belong to West African taurine cattle, populations 4 and 6 belong to West African zebu cattle, and populations from 6 to 8 belong to sanga cattle.

From a geographical point of view (Figure 4), the projection of admixture coefficients (Plots 4A and 4B) and the two second factors computed on both microsatellites and SNPs (Plots 4E and 4F) mirror the existence of two different genetic backgrounds (taurine and zebu cattle) in the sample, the introgression of zebu genes westwards and southwards, and a weaker differentiation between the N’Dama cattle of Burkina Faso and sanga and zebu cattle when compared with the N’Dama of Congo and the Lagunaire cattle. The two first factors 1 (Plots 4C and 4D) mainly reflect the differentiation by distance from the zebu cattle area. However, on the SNP dataset, the Lagunaire cattle departs from this pattern and can be easily identified (Plot 4D). The two third factors identified on both the microsatellite and SNP sets (Plots 4G and 4H) mainly mirrored the local genetic events: the contrast between the Lagunaire background and either that of the two zebu populations (Plot 4G) or N’Dama cattle (Plot 4H).

## 4. Discussion

The advantages of SNP arrays over microsatellites can be summarized as follows [1]: (a) much smaller confidence intervals around diversity measures allow better distinction between populations; (b) clustering methods showed a dramatic increase in the power to separate individuals into distinct groups; and (c) SNP data allow for complex questions such as local adaptation or evolutionary independence to be addressedthat cannot be considered with microsatellite markers (or any neutral loci) only. 

In the wild, it is possible to find population genetics studies reporting that microsatellites have comparable (and even better) performance than SNPs. However, these studies typically use a low number of bi-allelic markers and, therefore, the performance of SNP sets cannot overcome that of the more polymorphic microsatellite markers [4]. In non-model species, in which the number of markers available can be low, microsatellites have been reported to give comparable results to SNPs. The number of SNPs typed must be at least 5-fold that of microsatellites to obtain comparable results in population genetics studies [3]. However, microsatellite sets including 17–20 markers have been useful to reveal sub-structuring within populations [5] and are a good proxy for genome-wide SNP diversity [25], even if the SNP sets include about ten thousand markers.

The size of the markers’ sets is not an issue in projects targeting most livestock populations: a large number of microsatellites have beendeveloped since the 1990s and the availability of SNP arrays including thousands of SNPs is, at present, the rule. This fact, together with knowledge on the livestock breeding scenarios worldwide, makes it possible to use them (here using West African cattle) to assess the differences in performance of microsatellites and SNPs in practical terms.

### 4.1. Structure of the Analyzed Population

In general, both microsatellites and SNPs allowed us to identify the same pattern of genetic structuring in the West African cattle analyzed: there are two well differentiated subpopulations formed by taurine (Lagunaire and N’Dama breeds) and zebu cattle, with sanga cattle taking intermediate positions between those main cattle types (Figure 2 and Figure 3). However, sanga cattle share a genetic background with zebu cattle to a higher extent than with taurine cattle (Figure 1 and Figure 2). This general scenario is consistent with expectations. Farmers’ decisions aiming at increasing the body size of livestock bred in tsetse challenged areas of West Africa, the increase of the duration of the dry seasons since the 1970s that is limiting the distribution of vectors of trypanosomosis, and intense livestock trading is promoting the apparition of almost continuous patterns of genetic and morphological livestock variation from the Sahel area southward [11,12,13]. However, the introgression of Sahelian zebu genes into the taurine cattle of Southern West Africa does not follow simple patterns and can depend on local agro-ecological features, making Sahelian cattle more suitable to be bred in humid areas of Southern Sahel [11]. Using microsatellites and various estimation methodologies, the Lobi and the N’Dama cattle of Burkina Faso analyzed here were previously shown to have non-negligible West African zebu admixture proportions (0.80 and 0.46, on average, respectively) [11]. This is consistent with the noticeable number of individuals of N’Dama of Burkina Faso showing admixture proportions typical of sanga, and even zebu, cattle (Figure 2). The case of the Lobi cattle used here is less clear due to the very limited sample available. The two Lobi individuals showed an intermediate position between taurine and zebu cattle (Figure 2). Lobi cattle (here considered sanga) are the Burkina Faso representatives of the Baoulé taurine cattle widely spread in West Africa and, although sanga cattle tend to be morphologically closer to West African zebu, they had morphological features resembling taurine cattle [12,13].

Microsatellites and SNPs differed, however, in identifying particular sources of variation within this general genetic scenario, namely the existence of two differentiated genetic backgrounds within the taurine cattle analyzed: Lagunaire and N’Dama. Microsatellites and SNPs gave highly correlated solutions for admixture proportions when onlythe existence of two parental populations was assumed (Table 2). It is clear that when forcing K = 2, a significant variation is ignored, therefore leading to similar resultsbeing obtained. However, SNPs were able to differentiate between Lagunaire and N’Dama cattle (Figure 2B). If differentiation between Lagunaire and N’Dama cattle only affected the Congo population, it could be explained by its different breeding history: isolated and free of the zebu gene introgression affecting the N’Dama cattle of Burkina Faso [11,12]. However, this did not happen and N’Dama cattle sampled in Congo and Burkina Faso tended to overlap (Figure 2B and Figure 3C). In any case, SNPs had enough informative ability to differentiate between the two N’Dama populations using the software ADMIXTURE when K was fitted to 5 (Figure 1B).

Differences between the Lagunaire and N’Dama genetic background were also suggested by PCA with Factor 2 (markers nature notwithstanding), separating the Lagunaire and the N’Dama individuals. Again, SNPs allowed a clear separation between the two N’Dama populations sampled, which could not be obtained using microsatellites (Figure 3B,D). This better performance of SNPs is not trivial. There is consensus that the definition of livestock populations in Africa does not follow the European concept of “breed” and between-populations genetic differentiation is likely to be due to geographic distance rather than to either type and morphology or expected different origin [8,11,26,27]. Although also separated by its breeding history, in our example, the N’Dama cattle assigned to the Fouta-Djallon region of Guinea would play the role of “isolated by distance” population [11]. However, “isolation by distance” was not strong enough to erase the influence of the Lagunaire cattle background. Even using microsatellites, Factor 1 identified using PCA differentiated Lagunaire cattle from the two N’Dama samples which, in turn, tended to overlap (Figure 3C).

### 4.2. Projection on Synthetic Maps

The number of SNPs used here greatly exceeded the size of microsatellite markers set. Therefore, it is not surprising that SNPs appeared as more suitable for detecting structuring. However, both marker types had comparable performance in the projection of genetic variability on geographic maps. This is important in practical terms because this strategy can beapplied to ascertain population structure and gene flow events when previous genetic information on the populations analyzed is not high [6,8].

Clustering analyses carried out forcing K = 2 gave comparable results (Figure 2; Table 2). However, PCA gathered more genetic variation and bidimensional dispersion plots gave different shapes per type of marker, which can lead to confusion (Figure 3). Inspection of Plots 3C and 3D would suggest the existence of three well defined taurine cattle populations, two of them (N’Dama cattle) weakly separated, and highly differentiated from sanga and zebu cattle which, in turn, would form a single population. In contrast, PCA-based plots constructed using a microsatellite polymorphism would suggest the existence of weakly differentiated, highly intermingled, subpopulations. However, projection of the factors identified on synthetic maps gave comparable images (Figure 4), allowing that interpretation does not go far beyond the results obtained. The sample typed here was previously analyzed using different approaches [11,12,13,26] and, therefore, there is a considerable amount of information allowing a correct interpretation of the scenarios depicted. However, this is not the rule.

Aside fromthe nature and number of loci, other factors may influence the performance of geographical analyses of genetic diversity in complex scenarios, namely habitat connectivity, sampling density, and the metrics used for the assessment of genetic relationships.

Habitat connectivity makes gene flow more or less difficult, leading to different correlations of allele frequencies [28]. Both isolated and bottlenecked populations usually give reduced genetic diversity. SNP arrays may fit better to scenarios of spatially smoothed allele frequencies due to isolation-by-distance [10]. This could be particularly true in scenarios with no clear restrictions for gene flow such as in the case of West African cattle [11,12,13,25] due to the higher ability of SNPs to identify sub-structuring populations [1,3]. However, in the current analysis, both SNP arrays and microsatellites were able to identify the same geographical spots of genetic variability (Figure 4) and, therefore, expected differences in habitat connectivity may not be the only reason to decide whether to use one type of marker or another.

Here, we have used well-known metrics (PCA eigenvector and admixture coefficients) to summarize genetic diversity because of their wide use in population genetics studies [6,8,26]. However, technical factors such as the genetic parameters used for projection or the density of sampling may affect the performance of the projection of genetic diversity on synthetic maps. In the wild, simple genetic parameters estimated at a population level such as expected heterozygosity and allelic richness have been useful in characterizing geographical areas acting as the source of genes (the so-called “abundant centers” or “hot spots” of variability) and distinguishing them from contact zones (the so-called “melting pots”) in which genetic variability results from the influence of various abundant centers [29,30,31]. However, when the differences in genetic signal are subtle, simple genetic parameters may not properly reflect the genetic differences betweenpossible abundant centers and contact zones and, therefore, the use of more complex metrics summarizing individual genetic information to assess the population’s diversity conditional to any other population may be advisable [9,26]. Software packages allowing for thecomputation of simple genetic parameters summarizing genetic diversity at a population level from SNP arrays information can be easily found [16]. However, the number of microsatellite-based user-friendly software allowing for complex statistics to be calculated such as contributions to diversity is even higher [32]. This may influence decisions on the type of markers to be used for a given project. Furthermore, projections of genetic diversity on synthetic maps tend to perform better, even if sample size per population is not high, when the number of geographical populations sampled is high and when gene sources and expansion patterns are clear [10,27,30].

## 5. Conclusions

The overall information provided contributes to give insights on whether either microsatellites or SNPs markers are useful for population genetics studies on structured populations. Large SNP arrays outperform microsatellite markers in identifying fine-scale population structuring due to a more precise assessment of the genotypic information of the individuals. However, for some practical termssuch as geographic studies of genetic variation, microsatellite markers sets can be still useful, particularly if research focuses on non-model organisms in which large-scale SNP arrays are not available or, simply, if either the funding or the availability of efficient hardware and software to handle such large datasets is limited. Furthermore, the current study clearly illustrates how fine-scale structuring can be difficult to interpret when not enough previous information on the history of the populations exists.

## Figures and Tables

**Table 1:** Description of sampling. The following information is given per population sampled: type of cattle into which the population (or breed) is classified, number of samples available (N; males in brackets), country, main location, and approximate latitude and longitude (in decimal degrees) in which sampling was carried out and agro-ecological areas into which these sampling areas were classified. Numbers attached to the names of the populations are consistent with those used in Figure 1, Figure 2 and Figure 3.

Cattle Sampling			Geographical Information				
Population	Type	N	Country	Location ^a^	Latitude	Longitude	Ecological Area
1. Lagunaire	Humpless shorthorn ^e^	33(6)	Benin	Plateau	8.023	2.511	Guinean
2. N’Dama (BF)	Humpless longhorn ^e^	47(12)	Burkina Faso	Comoé	9.901	−4.365	Sudano-Guinean
3. N’Dama (C) ^b^	Humpless longhorn	25(4)	Congo	Bas-Congo	11.303 ^b^	−2.272 ^b^	Sudano-Guinean
4. Zebu Peul (Be)	West African zebu ^f^	12(2)	Benin	Alibori	11.121	2.960	Sahel and Sudan-Sahel
5. Zebu Peul (BF)	West African zebu	32(19)	Burkina Faso	Dori	14.072	−1.573	Sahel
6. Borgou	Sanga ^g^	28(0)	Benin	Collines	9.901	2.511	Sudan-Sahel
7. Lobi ^c^	sanga	2(1)	Burkina Faso	Poni	10.308	−3.174	Sahel
8. Zou ^d^	sanga	6(0)	Benin	Zou	8.153	2.013	Guinean

^a^ Province or Department; ^b^ the N’Dama (C) population derived from two N’Dama bulls and 40 N’Dama heifers imported from the Fouta-Djallon region of Guinea; therefore, latitude and longitude used for the N’Dama (C) population corresponded to that of the Fouta-Djallon region of Guinea; ^c^ Burkina Faso representative of the Baoulé cattle; ^d^ Lagune X zebu crosses with different degrees of zebu admixture; ^e^ *B. taurus* cattle; ^f^ *B. indicus* cattle; ^g^ ancient hybrids of indigenous African *B. taurus* and *B. indicus* cattle.

**Table 2:** Pearson (product-moment; below diagonal) and Spearman (rank; above diagonal) correlation between individual admixture coefficients ($\hat{q}$) computed for K = 2 and eigenvectors corresponding to the first three factors retained using PCA using microsatellite and SNP polymorphism.

Markers Type		Microsatellites				SNPs			
		$\hat{q}$	Factor 1	Factor 2	Factor 3	$\hat{q}$	Factor 1	Factor 2	Factor 3
Microsatellites	$\hat{q}$		−0.638	0.880	−0.837	0.947	0.942	−0.942	0.003 **^ns^**
	Factor 1	−0.743		−0.697	0.418	−0.607	−0.609	0.604	0.584
	Factor 2	0.912	−0.730	1	−0.755	0.844	0.844	−0.836	−0.100 **^ns^**
	Factor 3	−0.696	0.176 *	−0.704		−0.841	−0.838	0.831	−0.179 *
SNPs	$\hat{q}$	0.978	−0.679	0.906	−0.765		0.987	−0.995	0.026 **^ns^**
	Factor 1	0.957	−0.606	0.893	−0.819	0.991		−0.983	0.043 **^ns^**
	Factor 2	−0.975	0.663	−0.905	0.780	−0.999	−0.992		−0.024 **^ns^**
	Factor 3	−0.136 **^ns^**	0.612	−0.076 **^ns^**	−0.514	−0.029 **^ns^**	0.085 **^ns^**	0.001 **^ns^**	

$\hat{q}$ were computed forcing K = 2; **^ns^** means that the correlation coefficient was not statistically significant (α = 0.05); * mean *p* < 0.05; all other correlation coefficients were statistically significant for *p* < 0.0001.

## Data Availability

The data presented in this study are available from the corresponding author on reasonable request.

## Supplementary Materials

The following information available online at https://www.mdpi.com/2076-2615/11/1/151/s1: Supplementary Figure S1:Ascertainment of the most likely number of K estimated using STRUCTURE and ADMIXTURE.
