# Supplementary material for: Population Structure Assessed Using Microsatellite and SNP Data: An Empirical Comparison in West African Cattle
Source: Animals (Basel). 2021 Jan 11;11(1):151. doi: 10.3390/ani11010151 (PMC7827059; doi:10.3390/ani11010151)

Supplementary Figure S1. Ascertainment of the most likely number of K estimated using STRUCTURE (Plot A) and Admixture (Plot B). In Plot A) ΔK was calculated, for the populations analysed, between K = 1 and K = 8 across 10 different runs per K as ΔK = mean(|L″(K)|) / sd(L(K)) as implemented in the website STRUCTURE HARVESTER. In Plot B) cross-validation errors for each K tested using the program ADMIXTURE v1.23 were computed splitting dataset into 5 folders for each K, being folders sequentially used as test sets while the other four were used for training.

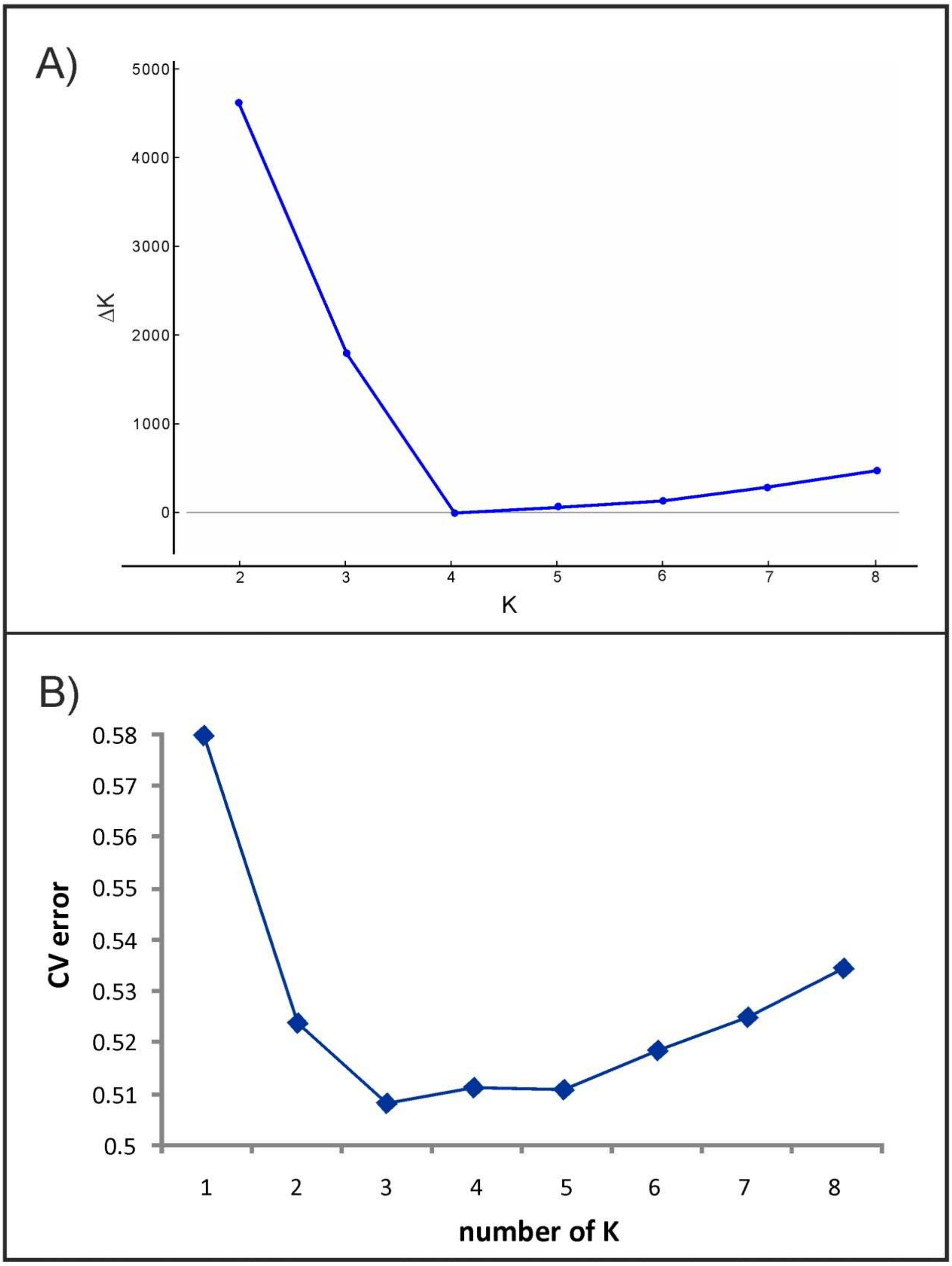

Supplement: Supplementary file 1 [file animals-11-00151-s001.pdf]
